# GP’s GP, general practitioner’s health and willingness to contract family doctors in China: a national cross-sectional study

**DOI:** 10.1186/s12875-024-02492-4

**Published:** 2024-07-12

**Authors:** Guoshu He, Jingjing Ren, Xiaoyang Chen, Qi Pan, Tianyuan Pan

**Affiliations:** Department of General Practice, The First Affiliated Hospital of Zhejiang University School of Medicine, Hangzhou, Zhejiang 310000 China

**Keywords:** Chronic condition, GPs, General practitioner, General practice, Doctors health, Family doctor, Self-treatment

## Abstract

**Objectives:**

General practitioners are trained to care for patients with a high level of responsibility and professional competency. However, there are few reports on the physical and mental health status of general practitioners (GPs) in China, particularly regarding help seeking and self-treatment. The primary aims of this study were to explore GPs’ expectations of their own family doctors and their reflection on role positioning, and to explore the objective factors that hinder the system of family doctors.

**Study design:**

Cross-sectional study.

**Methods:**

We conducted an online survey of Chinese GPs. Descriptive statistics were used to summarize the findings.

**Results:**

More than half of the participants (57.20%) reported that their health was normal over the past year. A total of 420 participants (23.35%) reported having chronic diseases. For sleep duration, 1205 participants (66.98%) reported sleeping 6–8 h per day; 473 participants (26.29%) reported chronic insomnia. Two hundred thirty-one participants (12.84%) had possible depression. A total of 595 (33.07%) participants reported that they had contracted a fixed family doctor. In terms of preventing themselves from contracting for a family doctor, the following factors were identified: lack of sufficient time (54.81%), could solve obstacles themselves (50.97%), and embarrassment (24.24%). The proportion of the contract group (12.44%) taking personal relationship as a consideration was higher than that of the non-contract group (7.64%) (χ2 = 10.934 *P* = 0.01). Most participants (79.90%) in the non-signed group reported never having seen a family doctor. In terms of obstacles, more than half of the signed group thought that they could solve obstacles themselves, while the non-signed group (39.20%) was less confident in the ability of family doctors than the signed group (29.75%) (χ2 = 15.436, *P* < 0.01).

**Conclusions:**

GPs work under great pressure and lack of self-care awareness, resulting in an increased prevalence of health conditions. Most GPs did not have a regular family doctor. Having a family doctor with a fixed contract is more conducive to the scientific management of their health and provides a reasonable solution to health problems. The main factors hindering GPs from choosing a family doctor were time consumption, abilities to solve obstacles themselves, and trust in the abilities of GPs. Therefore, simplifying the process of family doctor visits, Changing the GPs' medical cognition, and strengthening the policy of GP training would be conducive to promoting a family doctor system that enhances hierarchical diagnosis and treatment. International collaboration could integrate GP health support into global healthcare system.

## Introduction

Global healthcare systems facing common challenges, such as doctor's health issues, stress management, and their illness behaviors. Despite differences in healthcare systems and cultural backgrounds, there are striking similarities in the pressures and health challenges faced by general practitioners (GPs) [1–3]. A large proportion of GPs suffer from burnout, mild depression, chronic illnesses and denial of their own health problems. And most GPs tend to self-treat.

Physicain health behaviors exert a direct influence on patient outcomes [4]. Current research indicates that many physicians resort to self-treatment for their health issues [5, 6], potentially delaying formal healthcare access [1, 7, 8] and increasing associated risks. And, self-treatment is more common among GPs than specialists [4]. Numerous studies across different countries have explored physician's health statuses and medical behaviors, revealing substantial variations attributable to diverse cultural backgrounds and healthcare systems [5, 6, 9]. In countries with well-defined step-care systems such as the UK, Australia, and New Zealand, GPs generally demonstrate higher health care awareness and are more likely to consult their own GPs, promoting adherence to preventive health behaviors [1, 10, 11]. Conversely, only 14% of doctors in Hong Kong consulted a GP [3], predominantly younger physicians or those with a non-Hong Kong University (HKU) medical degree. Specialists, other than those in general practice, are less likely to seek GP services [3]. Research from Germany indicates that the majority of GPs possess private health insurance and most family doctors are also self-employed. The GP is usually (but not necessarily) the patient's first point of contact. Gps are chosen because they have a wide range of medical knowledge, covering many different specialities [2, 12]. China’s family doctor system, which differs from that in other countries, does not strictly adhere to a “first visit GP system”. Patients may choose between consulting a GP or a specialist directly. Nonetheless, China's policy promotes the Family Doctor Contract Services [13], with public health insurance favoring GP services and encouraging contracts with family doctors. Such contracts cover not only consultation fees, but also preventive checkups, including lifestyle guidance, vaccinations, and specialist referrals [14].

While patient care and healthcare systems have been extensively studied globally, there are clear gaps in the literature on personal health management by GPs themselves, particularly in non-Western countries. Most existing research has focused on patient outcomes and system efficiency, ignoring how GPs manage their own health needs under the pressures of their role. Given the direct impact that the well-being of GPs has on the quality of patient care and the sustainability of the healthcare system, this oversight is essential. Our research addresses this gap by exploring the health behaviours, challenges and support systems of GPs in China, a major non-Western country with a unique healthcare system. In doing so, it provides valuable insights into how to better support the health of GPs, contributing to the broader discussion about improving the quality of healthcare worldwide.

## Methods

### Study design

We conducted an online survey among GPs practicing in general practice in China. A questionnaire was developed based on a literature review and exploratory qualitative study. The questionnaire contained three sections: a sociodemographic section, questions that focused on GPs’ physical and mental health status, and questions focused on GPs' willingness to contract with family doctors. The survey was distributed through WeChat, the most popular social chat app among Chinese people.

### Procedures and data collection

Eligible GPs received a study information sheet, informed consent form, questionnaire, and assurance that their responses would remain anonymous. They were informed that their participation was completely voluntary. We designed a comprehensive questionnaire and adjusted it after conducting a pilot test with eight GPs. We asked the participants to sign the consent form and complete and return the questionnaire within four weeks.

### Eligibility

To take part in the survey, participants had to be a GP practicing in China, who were GP-qualified in China and provide the sighned consent form. Participants who accessed the survey from outside of China or who were still not in full practice (e.g., still in clinical rotation or training, interns, and trainee students), were excluded.

### Data analysis

Descriptive statistics were used to summarize the findings. Continuous variables were expressed as median and interquartile range (IQR), and categorical variables as frequencies and percentages. The χ^2^ test was performed to investigate whether there were group differences.

## Results

### Respondents

**Fig. 1:**
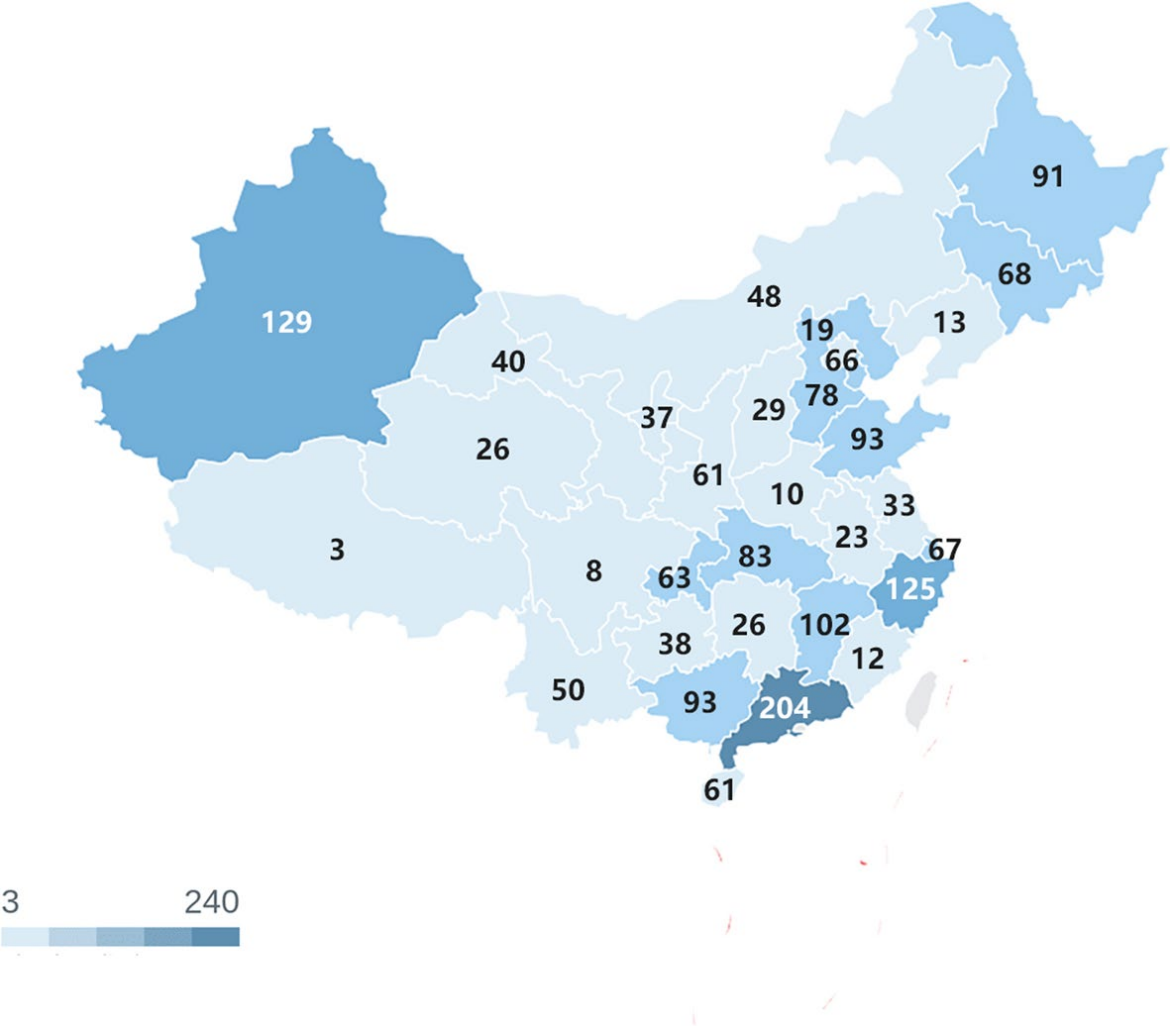
Geographic distribution of responding GPs (*n* = 1799)

Responses from 1808 participants were recorded. Of these, 9 were excluded because they did not indicate consent to take part in the survey. The remaining 1799 responses were used in the analysis. Responses were obtained from GPs from almost all the provinces in China (Fig. 1).

The age distribution was mainly concentrated between the ages of 26 and 50. The number of female participants (60.92%) was higher than the number of male participants (39.08%). Educational background showed that the most common background was a bachelor's degree (66.76%). The main workplaces were community health service centers (36.46%) and tertiary hospitals (33.69%). The training modes were mainly residence training (5 + 3) (30.62%) and transfer training(40.19%). The practice registration category was mainly western medicine (80.66%). The top five provinces by region were Guangdong, Xinjiang, Zhejiang, Jiangxi and Shandong (Table 1).

**Table 1:** Responents sociodemographic information (*n* = 1799)

	n	(%)
Age in years		
18–25	87	4.84
26–30	379	21.07
31–40	579	32.18
41–50	512	28.46
51–60	232	12.9
> 60	10	0.56
Gender		
Female	1096	60.92
Male	703	39.08
Educational background		
Technical secondary school	13	0.72
Junior college	132	7.34
Bachelor's degree	1201	66.76
Master degree or above	453	25.18
Work place		
Community health service center	656	36.46
Township health center	399	22.18
County-level hospital	58	3.22
Secondary hospital	80	4.45
Tertiary hospital	606	33.69
Training mode		
Residential training(3 + 2)	228	12.67
Residential training(5 + 3)	551	30.63
Transfer training	723	40.19
Untrained	208	11.56
Other	89	4
Category of practice registration		
Western medicine	1451	80.66
Integration of traditional Chinese and western medicine	67	3.72
Traditional Chinese medicine	106	5.89
Other	175	9.73
Province where main practice is located		
Guangdong	204	11.34
Xinjiang	129	7.17
Zhejiang	125	6.95
Jiangxi	102	5.67
Shandong	93	5.17
Other	1146	63.70

### Patterns of physical and mental health status

More than half of all the participants (57.20%) reported that they described their health as normal over the past year. When asked about their family relationships, most of them (60.20%) agreed they were harmonious with no pressure. Similar results were found in the responses to questions about interpersonal relationships in the work environment, in which more than half of the participants (51.81%) agreed that they get along well with colleagues and patients. Regarding eating habits, 1297 participants (72.10%) reported consuming a combination of meat and vegetables. However, almost half of the participants (44.41%) reported that their frequency of exercise was less than once a week, and methods of exercise varied. The participants reported various results regarding their enthusiasm for traveling. In addition, 410 participants (22.79%) said that they had never travelled, but when it came to choosing travel companions, most chose to travel with family members or friends instead of traveling alone. Among them, 88.99% never smoked, 45.3% never drank, and 40.36% drank occasionally (< once per month) (Table 2).

**Table 2:** Responents physical and mental health status

	n	(%)
How would you describe your health over the past year?		
Good	575	31.96
Normal	1029	57.20
Not good	165	9.17
Very bad	30	1.67
Your family relationship is very harmonious, and there are few conflicts with your family members.		
Agree	1083	60.20
Slightly agree	589	32.74
Not agree	108	6
Uncertainty	19	1.06
Your interpersonal relationships are harmonious and there is no pressure to get along with colleagues or patients.		
Agree	932	51.81
Slightly agree	676	37.58
Not agree	152	8.45
Uncertainty	39	2.17
Your eating habits		
Vegetarian dishes > 50%	300	16.68
Meat dishes > 50%	202	11.23
Combination of meat and vegetables	1297	72.10
Frequency of exercise		
At least once a week	595	33.07
Less than once a week	799	44.41
No habit of exercise	405	22.51
The usual way of exercise		
Running	702	39.02
Body-building	273	15.18
Swimming	142	7.89
Other	820	51.14
The habit of traveling		
Never travel	410	22.79
Less than once every two years	686	38.13
Once a year	493	27.40
More than twice a year	186	10.34
Once a month	21	1.17
Once a week	3	0.17
Do you travel with someone?		
Never travel	344	19.12
Alone	41	2.28
With friends	276	15.34
With family members	652	36.24
With friends and family members	486	27.02
The habit of smoking		
Never smoked	1601	88.99
1–5 cigarettes/day	78	4.434
5–10 cigarettes/day	39	2.17
10–20 cigarettes/day	64	3.56
20–30 cigarettes/day	13	0.72
> 30 cigarettes/day	4	0.22
How often do you drink?		
Never drink	810	45.03
Drink occasionally (< once a month)	726	40.36
Once a month	52	2.89
Once a week	106	5.89
Drink regularly (> once a week)	97	5.39
Drink every day	8	0.44
Do you currently have a chronic disease?		
Yes	420	23.35
No	1379	76.65
The chronic disease severity assessed by the doctor		
No problem	51	12.14
Mild	247	58.81
Moderate	108	25.71
Serious	13	3.1
Extremely serious	1	0.24
How many hours do you sleep everyday?		
< 4 h	9	0.5
4–6 h	518	28.79
6–8 h	1205	66.98
> 8 h	67	3.72
Do you often suffer from insomnia?		
Yes	473	26.29
No	1326	73.71
Do you take any medication for insomnia?		
Yes	141	29.81
No	332	70.19
PHQ-2		
Not depressive (0–2 points)	1568	87.16
Possibly depressive (3–6 points)	231	12.84

**Fig. 2:**
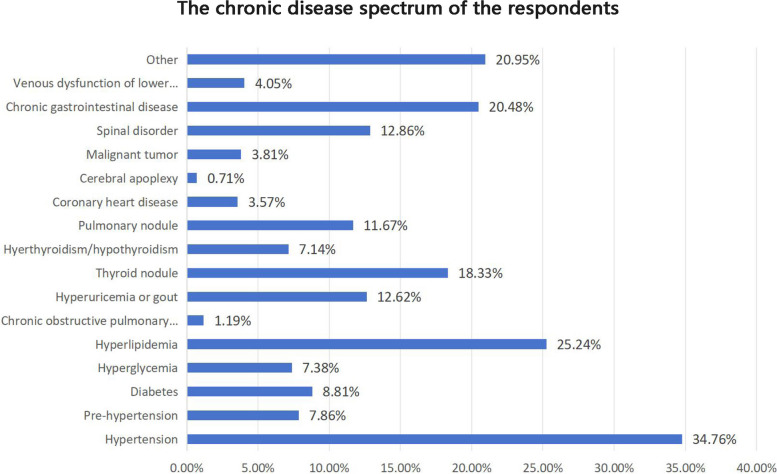
The chronic disease spectrum of the respondents (*n* = 420)

Four hundred and twenty participants (23.35%) reported having chronic diseases. The top three chronic diseases were hypertension (34.76%), hyperlipidemia (25.24%), and chronic gastrointestinal disease (20.48%) (Fig. 2). In terms of sleep, 1205 respondents (66.98%) participants reported sleeping 6–8 h per day, 518 (28.79%) reported sleeping 4–6 h per day, 9 (0.5%) reported sleeping less than 4 h per day, and 67 (3.72%) participants reported sleeping for more than 8 h per day. A total of 473 (26.29%) participants reported chronic insomnia. Among those with insomnia, 141 (29.81%) participants needed medication to help them sleep. The problem of depression among GPs should not be underestimated, with 231 participants (12.84%) possibly having depression.

### Contracting a regular family doctor

A total of 595 (33.07%) participants reported that they had contracted a fixed family doctor. We found no statistically significant associations between these varibles in those with chronic conditions. The two groups of participants, with and without a regular family doctor, had the same expectations of family doctors in the following aspects. They hoped that family doctors would have high professional skills (96.55%), high professional vision (45.91%), and high education (28.9%). They hoped that their family doctor would address the following issues: common illnesses (74.32%), disease assessment and prevention (69.98%), chronic conditions management (59.31%). In terms of preventing them from contracting for a family doctor, the following factors were identified: lack of sufficient time (54.81%), could solve obstacles themselves (50.97%), and embarrassment (24.24%) (Table 3).

**Table 3:** Variables related to the presence of chronic conditions and illness behaviour variables depending on contracted a fixed family doctor

	Contracted a fixed family doctor(*n* = 595), M (SD)/n (%)	Not contracted a fixed family doctor(*n* = 1204), M (SD)/n (%)	Comparison test
Chronic condition			
Yes	147(24.71)	273 (22.67)	χ^2^ = 0.918 (*P* = 0.338)
No	448(75.29)	931 (77.33)	
Do you think it is necessary for general practitioners to have a contracted family doctors?			
Yes	549 (92.27)	927 (76.99)	χ^2^ = 63.078 (*P* < 0.01)*
No	46 (7.73)	277 (23.01)	
Would you like to become a contracted family doctor for your colleague or general practitioner?			
Yes	551 (92.61)	884 (73.42)	χ^2^ = 90.795 (*P* < 0.01)*
No	44 (7.39)	320 (26.58)	
What do you think your contracted family doctor should have?			
High professional skills	577 (96.98)	1160 (96.35)	χ^2^ = 0.474 (*P* = 0.491)
Higher education	167 (28.07)	353 (29.32)	χ^2^ = 0.304 (*P* = 0.582)
Greater social influence	243 (40.84)	429 (35.63)	χ^2^ = 4.618 (*P* = 0.032)*
High professional field visibility	270 (45.38)	556 (46.18)	χ^2^ = 0.103 (*P* = 0.748)
Other	27 (4.54)	50 (4.15)	χ^2^ = 0.144 (*P* = 0.704)
What kind of family doctor would you choose for your family?			
Myself	253 (42.52)	398 (33.06)	χ^2^ = 15.447 (*P* < 0.01)*
Collegues	115 (19.33)	177 (14.70)	χ^2^ = 6.269 (*P* = 0.012)*
A general practitioner at a superior hospital	177 (29.75)	500 (41.53)	χ^2^ = 23.546 (*P* < 0.01)*
Other specialists in superior hospitals	45 (7.56)	112 (9.30)	χ^2^ = 0.560 (*P* = 0.454)
Other	5 (0.84)	17 (0.08)	χ^2^ = 1.077 (*P* = 0.299)
Who is your family doctor?			
Myself	255 (42.86)	683 (56.73)	χ^2^ = 30.701 (*P* < 0.01)*
Colleagues at the same hospital	247 (41.51)	194 (16.11)	χ^2^ = 138.833 (*P* < 0.01)*
A general practitioner at a superior hospital	81 (13.61)	267 (22.18)	χ^2^ = 18.713 (*P* < 0.01)*
Other specialists in superior hospitals	12 (2.02)	60 (4.98)	χ^2^ = 9.121 (*P* = 0.03)*
As a general practitioner, what is your main considerations for choosing your own family doctor?			
Professional technology	496 (83.36)	1073 (89.12)	χ^2^ = 11.841 (*P* = 0.01)*
Personal relationship	74 (12.44)	92 (7.64)	χ^2^ = 10.934 (*P* = 0.01)*
Other	25 (4.20)	39 (3.24)	χ^2^ = 1.075 (*P* = 0.30)
How often have you consulted your family doctor in the last 12 months?			
Never	270 (45.38)	962 (79.90)	χ^2^ = 219.876 (*P* < 0.01)*
1–2 times	239 (40.17)	181 (15.03)	χ^2^ = 140.577 (*P* < 0.01)*
3–4 times	37 (6.22)	28 (2.33)	χ^2^ = 17.328 (*P* < 0.01)*
5–6 times	14 (2.35)	7 (0.58)	χ^2^ = 10.832 (*P* = 0.01)*
> 6 times	35 (5.88)	26 (2.16)	χ^2^ = 16.848 (*P* < 0.01)*
In what ways do you seek help from your family doctor?			
Formal consultation	192 (32.27)	332 (27.57)	χ^2^ = 4.251 (*P* = 0.039)*
Simple consultation	295 (49.58)	647 (53.74)	χ^2^ = 2.76 (*P* = 0.097)
Only prescriptions or tests were required	87 (14.62)	120 (9.97)	χ^2^ = 8.474 (*P* = 0.004)*
Other	21 (3.53)	105 (8.72)	χ^2^ = 16.478 (*P* < 0.01)*
How do you deal with your common acute illness?			
self-diagnosis and treatments	310 (52.10)	618 (51.33)	χ^2^ = 0.095 (*P* = 0.758)
Consult your own family doctor	102 (17.14)	70 (5.81)	χ^2^ = 59.106 (*P* < 0.01)*
Consult other specialist in the same hospital	122( 20.50)	375 (31.15)	χ^2^ = 22.555 (*P* < 0.01)*
Consult specialists in other hospitals	61 (10.25)	141 (11.71)	χ^2^ = 0.850 (*P* = 0.356)
What problems would you most like your family doctor to solve for you?			
Common diseases	439 (73.78)	898 (74.58)	χ^2^ = 0.135 (*P* = 0.714)
Health assessment and disease prevention	412 (69.24)	847 (70.35)	χ^2^ = 0.232 (*P* = 0.630)
Chronic conditions management	334 (56.13)	733 (60.88)	χ^2^ = 3.717 (*P* = 0.054)
Mental health problems	325 (54.62)	678 (56.31)	χ^2^ = 0.461 (*P* = 0.497)
Other	20 (3.36)	34 (2.82)	χ^2^ = 0.395 (*P* = 0.530)
What do you think are the factors that prevent you and your family members from going to the family doctor?			
Awkwardness	149 (25.04)	287 (23.84)	χ^2^ = 0.315 (*P* = 0.575)
Lack of sufficient time	320 (53.78)	666 (55.32)	χ^2^ = 0.378 (*P* = 0.538)
Economic loss	106 (17.82)	233 (19.35)	χ^2^ = 0.615 (*P* = 0.433)
I think I can handle it	323 (54.29)	594 (49.34)	χ^2^ = 3.905 (*P* = 0.048)*
Don't trust the abilities of family doctors	177 (29.75)	472 (39.20)	χ^2^ = 15.436 (*P* < 0.01)*
Other	12 (2.02)	34 (2.82)	χ^2^ = 1.041 (*P* = 0.308)

^*^Stands for *P* < 0.05 (Statistically significant)

Two hundred forty-three (40.84%) participants with regular contract family doctors wanted family doctors to have a greater social impact. The group with regular contracted GPs was more likely to choose themselves (42.52%) and their colleagues (19.33%) as family doctors, whereas the group without contracted GPs was more likely to choose GPs from superior hospitals (41.53%) and themselves (33.06%). Among the family doctors who signed the contract, the signing group was mainly themselves (42.86%) and their colleagues (41.51%), and the non-signing group was themselves (56.73%) and GPs of superior hospitals (22.18%). In addition, a small number of uncontracted GPs (4.98%) chose specialists from superior hospitals. Among the factors to be considered in choosing a family doctor, most participants in both groups thought that professional skills were the most important especially those in the non-contract group (89.12%) when compared with those in the contract group (83.36%) (χ2 = 11.84, *P* = 0.01). The proportion of the contract group (12.44%) that took personal relationship as a consideration was higher than that of the non-contract group (7.64%) (χ2 = 10.934, *P* = 0.01). Most participants (79.90%) in the non-signed group reported never having seen a family doctor. Participants with a regular family doctor consulted their doctor significantly more often. In terms of the mode of consultation, contracted GPs preferred formal consultations (32.27%). However, among the non-signed group, a small number of participants (8.72%) indicated that there were other methods, in this regard further qualitative research is needed. More than half of the participants in both groups felt able to self-treat their acute illnesses. However, the signed group (17.14%) was more likely to consult their family doctor than the non-signed group (5.81%) (χ2 = 59.106, *P* < 0.01). By contrast, the uncontracted group was more likely to consult a specialist at the same hospital. However, approximately 10% still chose to consult a specialist at another hospital. In terms of obstacles, more than half of the signed group thought that they could solve obstacles themselves, while the non-signed group (39.20%) was less confident in the ability of family doctors than the signed group (29.75%) (χ2 = 15.436, *P* < 0.01) (Table 3).

## Discussion

### Innovation of the study

This study is a cross-sectional survey covering almost all the provinces in China. It has made significant progress in understanding the health behaviors of general practitioners (GPs) in China, setting a precedent for its application on a global scale. It uniquely addresses an often overlooked aspect of GP self-care by combining quantitative data with a comprehensive policy-oriented discussion, which is rare in the existing literature. By focusing on the specific health needs and behaviours of GPs and making tailored policy recommendations, our research fills a critical gap. It not only sheds light on common challenges faced by healthcare professionals worldwide, but also pioneers practical solutions that can be applied to different healthcare systems, strengthening the global discourse on the well-being of healthcare providers.

### Main findings of the study

Currently, despite the extensive training of a large number of GPs in various formats [15], both the quality and quantity of GPs fall short of expectations. This discrepancy results in GPs facing substantial workload [16, 17]. The physical and mental well-being of these practitioners is often overlooked [17, 18]. Although most GPs perceive their health as normal, many actually suffer from chronic conditions. Similar to studies in other countries, doctors tended to deny their own illnesses [1]. There are reasons for professional confidence, but also the desire to project a healthy and strong image in front of patients [4]. In terms of lifestyle, most GPs maintain healthy eating habits and abstain from tobacco and alcohol. However, they frequently neglect sufficient physical activity, as indicated by low exercise frequency. If GPs do not adequately address the psychological pressures of their role, through exercise, leisure activities, and work-life balance, they may face significant health challenges, such as depression [19]. Consequently, it is imperative that GPs have their own regular family doctors for ongoing health management [20, 21]. Fortunately, the mental health of GPs is better than that of specialists [6].

Most GPs did not have their own regular family doctor which had much to do with their understanding of the role and trust in the abilities of the family doctor [22, 23]. Many GPs resolve their health issues through self-diagnosis, a practice common in various countries [5], but criticized for its lack of objectivity and susceptibility to bias. Studies indicate that doctors who engage with a GP were more likely to receive preventive care [10, 24]. These doctors were three times more likely to visit a doctor for health maintenance than those who did not have a family doctor [11]. So GPs must adjust their ideas accordingly. Family doctors must address the problems of common diseases in patients, early screening for diseases, chronic disease management, and mental health problems. This study showed that GPs who had their own regular contracted family doctor consulted their doctor more frequently, and they would choose the formal method of consultation, which was also more conducive to their own health management [25].

When choosing a contracted family doctor, the convenience of medical treatment and personal rapport would also be considered [26, 27]. This finding is consistent with previous studies [9, 28]. Distrust in the abilities of GPs is an important aspect that hinders the advancement of the family doctor system [25]. Participants reflected that time factor would affect their choice; therefore, simplifying the process would be conducive to promoting the family doctor system. Governments utilizing electronic information technology could ease access to GP services, while the efficient management of electronic records could improve the family doctor system effectiveness. The results of our study showed that 50.97% of respondents believed that they could solve their health problems independently. However, this perception must be corrected. Based on the experience of other countries, efforts to inculcate more rational help-seeking behaviour should probably start in medical schools [7].

Financial factor, as one of the obstacles to signing contracts, also accounted for 18.84 percent. Although not one of the main factors, it also reflects a certain situation. The Chinese government has significantly reduced healthcare cost over the past decade, particularly through initiatives like the Family Doctor Contract Services, which have made healthcare more affordable [14, 29, 30]. However, costs remain a complex barrier, encompassing direct and indirect expenses such as fees, lost wages, and business insurance access, even among doctors who typically belong to higher socioeconomic groups [1].

Our study revealed some surprising results. Doctors are also well aware that GP has a greater role to play in the prevention and management of chronic conditions than dealing with the acute illnesses. But interestingly, our results showed no significant difference between the contracted group and the non-contracted group with chronic conditions. The reasons for this need to be further explored.

### Policy implications to global healthcare systems

Our study underscores the need for robust health support systems tailored to GPs, applicable both in China and globally. Recognizing universal challenges like stress and self-treatment among GPs, we advocate for structured support programs that include regular health assessments and mental health services specifically designed for medical professionals. Policymakers should also introduce incentives for GPs to engage more actively in their own health care, such as subsidizing healthcare costs or offering dedicated services, alongside establishing mandatory, regularly scheduled health checks.

### Call for international collaboration

Furthermore, there is a critical need for international collaboration in developing policies that support GP health, sharing successful strategies and outcomes to craft effective support mechanisms adaptable across different healthcare systems. For instance, benchmarking systems that have successfully integrated GP health support into their healthcare policies could serve as models for others to follow [31, 32]. By integrating these policy recommendations into national and international health agendas, we can enhance the sustainability of healthcare systems and the well-being of GPs worldwide, ultimately improving the overall quality of healthcare delivery.

#### Strengths and limitations

The study was innovative in exploring from the perspective of GPs their reasons for not signing up for family doctors, and analyzing their responses regarding the capabilities that family doctors need to have. It uniquely addresses an often overlooked aspect of GP self-care by combining quantitative data with a comprehensive policy-oriented discussion, which is rare in the existing literature. However, China is a vast country with an uneven distribution of GPs, and their number varies from region to region; therefore, there are limitations to conducting such a large-scale survey at the national level. The participants were distributed all over the country but the sample distribution was biased. Therefore, more rigorous and stratified sampling studies are required. As the survey was anonymized and cross-sectional, no further data could be tracked. In addition, some answers from non-signed doctors need to be further explored through qualitative research methods.

## Conclusions

General practitioners work under great pressure, experience mental tension, and lack self-care awareness, resulting in an increased prevalence of health conditions. Most GPs thought they had normal health but, in fact, they suffered from chronic diseases and were not optimistic. Most GPs did not have a family doctor. Having a family doctor with a fixed contract is more conducive to the scientific management of their health and provide a reasonable solution to health problems. GPs’ expectations of family doctors include higher clinical professional abilities, comprehensive professional knowledge, and higher educational background. The main factors hindering GPs from choosing family doctors were time consumption, abilities to solve obstacles themselves, and trust in the abilities of GPs. Therefore, simplifying the process of family doctor visits, Changing the GPs’ medical cognition, and strengthening the policy of GP training are conducive to promoting a family doctor system that enhances hierarchical diagnosis and treatment. International collaboration could integrate GP health support into global healthcare system.

## Data Availability

No datasets were generated or analysed during the current study.
